# Retrospective analysis of 104 histologically proven adult brainstem gliomas: clinical symptoms, therapeutic approaches and prognostic factors

**DOI:** 10.1186/1471-2407-14-115

**Published:** 2014-02-21

**Authors:** Thomas Reithmeier, Aanyo Kuzeawu, Bettina Hentschel, Markus Loeffler, Michael Trippel, Guido Nikkhah

**Affiliations:** 1 Department of Neurosurgery, Schwabing Academic Teaching Hospital, Munich, Germany; 2 Service de Neurochirurgie, Hopital Louis Pasteur, Colmar, France; 3 Institute for Medical Informatics, Statistics and Epidemiology, University of Leipzig, Leipzig, Germany; 4 Division of Stereotactic Neurosurgery, Department of General Neurosurgery, University Freiburg – Medical Centre, Freiburg, Germany; 5 Department of Neurosurgery, University Hospital, Erlangen, Germany

**Keywords:** Brainstem glioma, Adult, Neuropathology, Stereotactic surgery

## Abstract

**Background:**

Adult brainstem gliomas are rare primary brain tumors (<2% of gliomas). The goal of this study was to analyze clinical, prognostic and therapeutic factors in a large series of histologically proven brainstem gliomas.

**Methods:**

Between 1997 and 2007, 104 patients with a histologically proven brainstem glioma were retrospectively analyzed. Data about clinical course of disease, neuropathological findings and therapeutic approaches were analyzed.

**Results:**

The median age at diagnosis was 41 years (range 18-89 years), median KPS before any operative procedure was 80 (range 20-100) and median survival for the whole cohort was 18.8 months. Histopathological examinations revealed 16 grade I, 31 grade II, 42 grade III and 14 grade IV gliomas. Grading was not possible in 1 patient. Therapeutic concepts differed according to the histopathology of the disease. Median overall survival for grade II tumors was 26.4 months, for grade III tumors 12.9 months and for grade IV tumors 9.8 months. On multivariate analysis the relative risk to die increased with a KPS ≤ 70 by factor 6.7, with grade III/IV gliomas by the factor 1.8 and for age ≥ 40 by the factor 1.7. External beam radiation reduced the risk to die by factor 0.4.

**Conclusion:**

Adult brainstem gliomas present with a wide variety of neurological symptoms and postoperative radiation remains the cornerstone of therapy with no proven benefit of adding chemotherapy. Low KPS, age ≥ 40 and higher tumor grade have a negative impact on overall survival.

## Background

Adult brainstem gliomas are a very rare (<2% of gliomas) and poorly investigated disease. Recently several larger series on patients with brainstem gliomas have been reported, however, these series were only partly based on a histologically established diagnosis (Kesari [1], Landolfi [2], Guillamo [3], Salmaggi [4]) or involved also other pathologies than gliomas (Rachinger [5], Samadani [6]). Importantly, Rachinger and colleagues recently stated that intraaxial brainstem lesions with a radiological pattern of glioma represent a very heterogeneous tumour group with completely different outcomes and that metastasis, lymphoma, inflammation and cavernoma could be misinterpreted as a glioma by magnetic resonance imaging (MRI).

The goal of this study was therefore to analyze clinical, prognostic and therapeutic factors in the largest series of histological proven brainstem glioma reported so far.

## Methods

### Patients and data collection

Between 1997 and 2007, 104 patients (age > 18 years) with histologically proven gliomas of the brainstem were included in this study from five German centres (Freiburg: n = 73, Tübingen: n = 12, Munich: n = 10, Dresden: n = 7, Bonn: n = 2). The brainstem was subdivided in a superior (mesencephalon, crus cerebri and lamina quadrigemina), middle (pons) and inferior part (medulla oblongata). The tumor was defined as a brainstem glioma when more than 50% of the tumor involved the brainstem and a histological diagnosis of a glioma was available. This definition includes according to Donaldson and Reith diffuse brainstem gliomas (brainstem involvement > 50%) as well as focal brainstem gliomas (brainstem involvement < 50%) and excludes tumors which significantly involve areas adjacent to the brainstem [7,8]. Data on clinical course of disease, neuroradiological imaging, therapeutic approaches and neuropathological findings were collected and analyzed with the assistance of the central database.

Tissue samples were available from all patients either by stereotactic biopsy or microsurgical tumor resection and clinical follow-up information was collected on electronic case report forms in regular intervals. The local ethical committees of the participating institutions (Freiburg, Tübingen, Munich, Bonn, Dresden) enrolling patients approved the study.

The local neuropathologist of the corresponding university centre enrolling a patient performed neuropathological diagnosis. Preoperative MRI examination was performed by university neuroradiologist in 47 cases and by local radiologist of the admitting institution in 57 cases. Neuroradiological findings of the preoperative MRI were analyzed for etiological classification. Central neuroradiological review was not performed to depict daily clinical practice.

### Statistical analysis

The association of clinical data was tested by χ2-test, Fisher’s exact test and Kruskal-Wallis-test. Logrank test was used to compare outcome data. Cox regression models for OS were fitted to assess the impact of age (<40 vs. ≥ 40), WHO grading (grade I+II vs. grade III+IV), KPS (≤70 vs. > 70) and initial treatment (no vs. external beam radiation or radiochemotherapy). Data were analyzed by IBM SPSS (Version 20.0.0) and StatXact-8 (Cytel Studio Version 8.0.0).

## Results

### Patient population

Patient characteristics are given in Table 1. All patients were adults and age ranged from 18 to 89 years (median 41 years). WHO grading was determined in 103 patients (grade I glioma in 15.5%, grade II glioma in 30.1%, grade III glioma in 40.8% and grade IV glioma in 13.6%). Median Karnofsky Performance Score (KPS) at diagnosis was 80 (range 20–100). The male to female ratio was 58.7% to 41.3%. Patients with high-grade tumors were more often male (p = 0.024) and older (p = 0.041) than patients with low-grade tumors. Median follow up of the whole population was 49.3 months. Median overall survival was 18.8 months with 95% CI from 11.2 to 26.3 months (1-year-OS-rate 60.9%, 2-year-OS-rate 44.1%, and 5-years-OS-rate 34.0%).

**Table 1:** Patient characteristics

	**All patients N = 104**
Age (years)	
Median (range)	40 (18 - 89)
Gender, n(%)	
Male	61 (58.7%)
Female	43 (41.3%)
KPS (n = 71)	
Median (Range)	80 (20 - 100)
≤ 70	26 (36.6%)
> 70	45 (63.4%)
Extend of resection	
Stereotactical biopsy	93 (89.4%)
Microsurgical operation	11 (10.6%)
Histopathological WHO-diagnosis (n = 101)	
Oligoastrocytoma II	1 (1.0%)
Anaplastic oligoastrocytoma III	1 (1.0%)
Ependymoma II	2 (2.0%)
Diffuse Astrocytoma II	23 (22.8%)
Anaplastic astrocytoma III	39 (38.6%)
Fibrillary astrocytoma	4 (4.0%)
Pilocytic astrocytoma	17 (16.8%)
Glioblastoma	14 (13.9%)
WHO grade (n = 103)	
Low grade	47 (45.6%)
High grade	56 (54.4%)
First-line treatment (n = 101)	
External beam radiation	45 (44.6%)
Radio-/Chemotherapy	22 (21.8%)
Interstitial radiosurgery	7 (6.9%)
Chemotherapy	4 (4.0%)
No tumor specific therapy	23 (22.8%)

### Initial symptoms

There was a wide variety of symptoms and combination of symptoms at the time of initial presentation. The most common presenting symptoms were sensory symptoms (29.8%), symptoms of cranial nerves II, III, IV and VI (ophthalmological symptoms in 28.8%), impaired coordination (28.8%), paresis (21.2%), pain (21.2%), gait ataxia (18.3%), dysarthria and dysphagia (13.5%), signs of raised intracranial pressure (12.5%), organic psycho-syndrome (7.7%), nausea and vomiting (6.7%), myoclonus (2.9%), tinnitus or auditory disturbances (1.9%), incontinence (1.9%). In 4.8% diagnosis of a brainstem glioma was an incidental finding. 40.4% of patients presented with one symptom, and 54.8% with a combination of up to 6 symptoms.

### Neuroradiological imaging

Preoperative T1-weighted MR images were available in 95 patients, T2-weighted images in 32, FLAIR sequences in 7, diffusion-weighted images in 5 patients and additional sequences were performed in 5 patients. Data about the location of the tumor were available in 99 cases. The tumor was located solely in the inferior brainstem in 6 cases (6%), in the middle brainstem in 33 cases (33%) and in the superior brainstem in 19 cases (19%). Two parts of the brainstem were involved in 41 cases (41%) with an infiltration of the inferior/middle brainstem in 23 cases (23%) and of the middle/superior brainstem in 18 cases (18%). Definitive diagnosis of a glioma by neuroradiological imaging was made in 41 cases, diagnosis of another disease was made in 7 cases and in 56 cases no conclusive diagnosis was made. Most common differential diagnoses were lymphoma (n = 4), inflammatory disease (n = 3), abscess (n = 3), metastasis (n = 2), demyelinating disease (n = 1), ependymoma (n = 1), hemangioblastoma (n = 1) and infarction (n = 1).

### Initial surgical procedure and complications

Tissue samples were obtained by stereotactic biopsy in 89.4% (93 patients). The majority of patients were operated in supine position with a frame-based stereotactic system in local anesthesia by a frontal approach. A suboccipital approach was chosen in 2 patients. Mean duration of the operative procedure was 93 minutes and an average of 7 probes was obtained.

In 10.6% (11 patients) a microsurgical operation was performed. Mean duration of the operative procedure based on 7 patients was 203 minutes, with a total resection in 2 patients, a subtotal resection in 2 patients, a partial resection in 1 patient and a biopsy in 5 patients. Data from one patient were missing.

The rate of postoperative complications was 11.8% (11 patients) in stereotactically biopsied patients. Severe complications occurred in 2.2% and consisted of acute coma and hemiparesis caused by occlusive hydrocephalus due to postoperative bleeding in one patient, and a postoperative pontine bleeding combined with an infarction in one patient. Other complications occurred in 9.6% (9 patients). 1 patient developed an aggravation of ptosis and double vision, in 1 patient a dysphagia, dysarthria and facial paresis occured, and 1 patient suffered from singultus. One patient developed a focal epilepsy and aggravation of preexisting hemiparesis and one patient an aggravation of dysarthria, dysphagia and ataxia. One patient developed an intracranial abscess and one patient a liquor leckage. In two patients clinical asymptomatic postoperative hemorrhage was detected in the postoperative CCT scan.

The rate of perioperative complications in the microsurgically operated group was 36.4% (4 patients) and consisted of 2 postoperative hemorrhages, 1 infection of the bone flap and 1 respiratory insufficiency.

### Histopathological results

103 of 104 tumors were graded according to the WHO classification and in 1 patient grading was not possible by the local universitary neuropathologist. 16 patients had a grade I, 31 patients a grade II, 42 patients a grade III and 14 patients a grade IV tumor.

Histopathological diagnosis according to the WHO classification was possible in 101 patients (Table 1). Anaplastic pilocytic astrocytoma was diagnosed in two patients and an astrocytoma without any further classification in another patient.

### Treatment

#### Initial treatment

Initial treatments after surgery were chemotherapy, external beam radiation, interstitial radiosurgery with implantation of I-125 seed, a combination of radio- and chemotherapy or a wait and see strategy. Information about all treatments administered was available in 101 of 104 patients. In 23 patients a wait and see approach was chosen, 22 patients received combined radiochemotherapy, 45 patients were treated with external beam radiation, 7 patients with interstitial radiosurgery and 4 patients with chemotherapy alone. Median overall survival for patients treated supportive was 4.3 months, for patients who received external beam radiation was 26.4 months and for patients treated by radio-/chemotherapy was 13.4 months. Therapeutic strategies differed between the different WHO grades. Brachytherapy was performed only in low-grade gliomas whereas radiochemotherapy was predominantly given to patients with high-grade gliomas (17 patients with high grade gliomas vs. 5 patients with low-grade gliomas; for details see Table 2).

**Table 2:** Therapeutic strategies according to WHO grade

	**WHO grade**				**Total**
	**I**	**II**	**III**	**IV**	
Therapy					
External beam radiation	6 (40.0%)	16 (53.3%)	18 (43.9%)	4 (28.6%)	44 (44.0%)
Radio-/Chemotherapy	1 (6.7%)	4 (13.3%)	12 (29.3%)	5 (35.7%)	22 (22.0%)
Interstitial radiosurgery	4 (26.7%)	3 (10.0%)	-	-	7 (7.0%)
Chemotherapy	-	-	2 (4.9%)	2 (14.3%)	4 (4.0%)
No tumor specific therapy	4 (26.7%)	7 (23.3%)	9 (22.0%)	3 (21.4%)	23 (23.0%)
Total	15 (100.0%)	30 (100.0%)	41 (100.0%)	14 (100.0%)	100 (100.0%)

Data on salvage treatment at progression were available in 22 patients. Chemotherapy alone was performed in 11 patients. 6 patients received temozolomide, and 5 patients received a combination of temozolomide and ACNU, temozolomide and PC, temozolomide and PCV, or PCV and ACNU alone. Radiotherapy was performed in 8 patients, a combination of radio- and chemotherapy in 1 patients, a combination of brachytherapy and chemotherapy in 1 patient, and brachytherapy alone in 1 patient.

#### Influence of KPS and age on treatment decision

35.6% of patients over 40 years received supportive therapy compared to 15.6% of patients < 40 years (p = 0.069). No tumor-specific therapy was initiated in 54.2% of patients with a KPS ≤ 70 compared to 8.1% of patients with a KPS > 70 (p<0.001).

### Overall survival

Median overall survival for the whole population was 18.8 months but differed significantly for the different WHO grades. Median overall survival for grade II tumors was 26.2 months, for grade III tumors 12.9 months and for grade IV tumors 9.8 months.

Treatment was associated with improved survival in the high-grade glioma as well as in the low-grade glioma group. Supportive care only was associated with an unfavourable outcome in both groups. In the high-grade group the addition of chemotherapy to radiotherapy was not associated with improved survival.

### Prognostic factors

**Figure 1:**
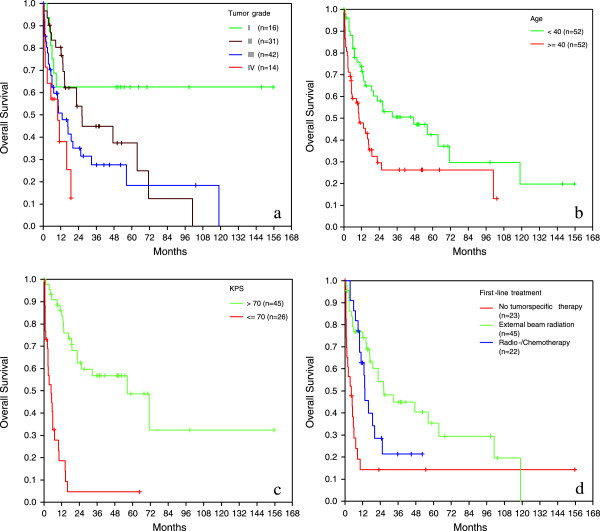
Prognostic factors: a) tumor grade; b) age, c) Karnofsky performance index, d) therapy related factors.

Grading correlated with survival (p = 0.003, Figure 1a). In univariate analysis high-grade tumors had an about two times increased relative risk (RR) for death related to low-grade tumors.

Younger age was associated with improved overall survival. Median overall survival for patients < 40 years was 47.3 months whereas patients ≥ 40 years had a median overall survival of 10.3 months (p = 0.006; Figure 1b) with an increased risk for death by factor 2.

Karnofsky performance score at diagnosis was associated with prognosis: patients with a KPS > 70 had a median overall survival of 56.3 months whereas patients with a KPS ≤ 70 had a median overall survival of 4.8 months (p<0.001, Figure 1c), with an increased risk of death by factor 7.1.

Initial treatment (supportive care = no tumor-specific therapy, external beam radiation, radiochemotherapy) was also associated with overall survival on univariate analysis: median overall survival for patients, who received no tumor-specific therapy was 4.3 months whereas patients who were initially treated by external beam radiation had a median overall survival of 26.4 months (external beam radiation vs. no tumor-specific therapy, p<0.001) and patients treated with a combined radiochemotherapy had a median overall survival of 13.4 months (radiochemotherapy vs. no tumor-specific therapy p = 0.003). There was no significant difference in overall survival among patients who received external beam radiation alone as opposed to radiochemotherapy (p = 0.093, Figure 1d). External beam radiation or radiochemotherapy reduces the risk to die by factor 0.3 and 0.4.

To assess the independent impact of the above mentioned factors on overall survival a multivariate cox regression model was built. Karnofsky performance score ≤ 70 showed the strongest effect and increased the relative risk to die by factor 6.7, followed by therapy (radio-chemotherapy reduced the relative risc to die by factor 0.3 and external beam radiation by factor 0.4). High grade glioma increased the relative risc to die by the factor 1.8 and age ≥ 40 by 1.7. However on multivariate analysis age and WHO grade did not reach statistical significance (Table 3).

**Table 3:** Cox regression models to assess the impact of age (<40 vs. ≥ 40), WHO grading (grade I + II vs. grade III + IV), KPS (≤70 vs. > 70) and initial treatment (no vs. external beam radiation or radiochemotherapy) on the relative risc to die

	**Relative risk**	**95% CI**	**p-value**
Age (years)			
< 40	1		
≥ 40	1.7	0.8 to 3.4	0.143
KPS			
> 70	1		
≤ 70	6.7	2.9 to 15.8	< 0.001
WHO grade			
Low	1		
High	1.8	0.9 to 3.6	0.104
Treatment			
No tumor specific therapy	1		
External beam radiation	0.4	0.2 to 0.9	0.021
Radio-/Chemotherapy	0.3	0.1 to 0.9	0.041

## Discussion

Here we report one of the largest series of histopathologically proven gliomas of the brainstem. Patients presented with a wide variety of symptoms, and median survival was only 18.8 months for the whole patient population. The amount of neuroradiological differential diagnoses confirmed the necessity of histopathological evaluation. In multivariate analysis KPS ≤ 70, higher tumor grade and age ≥ 40 were negative prognostic factors, whereas radiation therapy or radio-chemotherapy improved prognosis.

Recently several large series about brainstem gliomas in adults with median overall survival rates between 54 and 85 months have been published (Table 4). However diagnosis of a brainstem glioma in these series was mainly based on neuroradiological imaging and confirmed by histopathological examinations only in 13% - 67% of cases. Rachinger found that in a series of 46 radiologically suspected brainstem gliomas histological examination confirmed a glioma in only 28 cases (61%) and revealed metastasis in 15% (n = 7), lymphoma in 11% (n = 5), inflammatory disease in 4% (n = 2), cavernoma in 2% (n = 1) and gliosis in 6% (n = 3). The authors pointed out, that intra-axial brainstem lesions with a radiological pattern of glioma represent a very heterogeneous tumor group with completely different clinical outcomes [5]. Samadani published a meta-analysis of 293 brainstem biopsies in children and adults. Stereotactic biopsy was in 96% diagnostic with a mortality rate of 0.3%, a transient morbidity rate of 4% and a permanent morbidity rate of 1%. Pathology showed that half of the adult brainstem intrinsic lesions were gliomas, 10% were metastases, and the remainders were hematomas, vascular malformations, lymphomas, demyelination, cysts, radiation necrosis, abscesses, vasculitis, infarcts, leukemia, cryptococcus, or granulomas. They pointed out that neuroradiological diagnosis of non-contrast-enhancing lesions of the brainstem as low grade glioma is insufficient and that histopathological diagnosis for non-enhancing brainstem lesions with a long duration of symptoms revealed hematomas, arteriovenous malformations, lymphomas, demyelination, radiation necrosis and infarction. Therefore the authors concluded that stereotactic biopsy is indicated for both enhancing and non-enhancing lesions of the brainstem [6].

**Table 4:** Literature overview of large series of adult brain stem glioma: histological confirmation of diagnosis varied between 13% and 100% (actual study)

**Author**	**N**	**Histology**	**HGG**	**LGG**	**Median OS**
Landolfi (1998)	23	3 (13.0%)	1 (33.3%)	2 (66.7%)	54.0 months
Salmaggi (2008)	34	20 (58.8%)	11 (55.0%)	9 (45.0%)	59.0 months
Guillamo (2001)	48	32 (67.0%)	15 (46.9%)	17 (53.1%)	64.8 months
Kesari (2008)	101	46 (45.5%)	31 (68.9%)	15 (31.1%)	85.0 months
Reithmeier (2013)	104	104^*^ (100.0%)	46 (44.7%)	57 (55.3%)	18.8 months

*In one patient grading was not performed.

Kickingereder et al. recently published a large meta analysis of 1480 stereotactic biopsies for brainstem tumors and found a diagnostic success rate of 96.2%, an overall morbidity rate of 7.8%, a permanent morbidity rate of 1.7% and a mortality rate of 0.9% [9].

The clinically relevant postoperative morbidity of 9.7% in our series is in line with the data of Kickengereder but higher in comparison with figures from the meta-analysis of Samadani and colleagues [6]. The main difference between these two series was the homogenous histopathology in our series which consisted only of gliomas with a proportion of high-grade tumors of nearly 50% as opposed to a broad variety of tumorous and non-tumorous diseases in the latter series. Because of the rich neovascularization of high-grade gliomas, the risk of of postoperative hemorrhage or malignant brain-edema is likely to be higher in malignant gliomas compared to other pathologies and may explain the differences in morbidity.

Several publication have discussed this issue with regard to stereotactic brain biopsy for supratentorial lesions. Bernstein suggested that biopsy of specific pathologies (e.g. glioblastoma, lymphoma) may be associated with an increased risk of either hemorrhage or severe edema, due to the abnormal neovasculature of these tumors [10]. Savin confirmed these data and identified malignant glioma pathology to be associated with a 4-fold increased risk of morbidity, especially from hemorrhage [11]. However other authors found no association between lesion pathology and complication rates [12]. A recent study about complication of frame-based stereotactic biopsy in 622 cases identified an association between mortality and glioblastoma pathology and suggested that abnormal tumor neovasculature of malignant glioma may be the reason therefore [13].

Overall morbidity of stereotactic biopsy in brainstem tumors ranges between 7.8% and 12% in larger series and is therefore not distinctly higher compared to a morbidity rate of 4.9% in general stereotactic brain biopsy, especially when considering the low rate of 1.7% of permanent morbidity in stereotactic brainstem biopsy [1,4,9,13].

Dellaretti and colleagues investigated the correlation between magnetic resonance imaging findings and histological diagnosis of intrinsic brainstem lesions in adults in a series of 96 patients. Stereotactic biopsy established a precise histological diagnosis in 92 patients which consisted of 63 diffuse brainstem gliomas, 19 other neoplastic diseases (lymphomas, metastases, pilocytic astrocytomas, craniopharyngioma, ganglioma) and 10 non-neoplastic lesions (inflammatory disease, ischemic lesion, fungal abscess, gliosis). Overall morbidity rate was 9% and one patient died from exacerbated peritumoral edema. With regard to neuroradiological features the diagnostic effect of stereotactic biopsy was greater in patients with focal or enhancing lesions shown by MRI in whom the diagnosis of a diffuse gliomas was less frequent [14].

The value of additional imaging modalities to improve non-invasive diagnostic accuracy by MR spectroscopy or positron emission tomography is currently under investigation. However, Massager showed recently in a series of 30 brainstem gliomas that the integration of PET imaging can not replace histological analysis as MRI combined with PET data was only concordant with histological findings in 63% of cases [15].

The results of these studies are indicative that in adult patients with lesions of the brainstem therapeutic decisions should be based on a histopathological examination due to the wide spectrum of differential diagnoses.

Therefore we included in our study only patients with a histopathologically confirmed brainstem glioma to exclude a possible bias due to non-glioma lesions classified as gliomas by MRI, which may have a significant better prognosis. This and the high rate of malignant brain stem gliomas of 44.7% might explain the distinct difference in median overall survival of 18.8 months in our series in comparison to the actual literature of brainstem gliomas. We found that median overall survival of treated patients with HGG of the brainstem resembled the overall survival data of patients with supratentorial high grade gliomas. However median overall survival of patients with grade II gliomas of the brainstem was significant shorter than in brainstem glioma series of Kesari (26.4 months vs. 168 months) and in comparison to supratentorial low grade gliomas (26.4 months vs. 7–8 years). Reasons for this difference might be the location within a highly eloquent area, faster malignant transformation than in supratentorial gliomas for unknown reasons or possible histopathological undergrading. It is also notable that in Kesari’s series grade I brainstem gliomas had a significant shorter median overall survival of 83 months in comparison to grade II brainstem gliomas with a median overall survival of 168 months. Inaccurate neuroradiological diagnosis and subsequent undergrading may also explain this as up to 60% of high grade brainstem gliomas show no contrast enhancement after gadolinium application [6].

The location within a highly eloquent area may also explain the finding that the percentage of glioblastoma is significant lower in the brainstem compared to its supratentorial counterpart (12.5% vs 60-75%) [16] as brainstem gliomas may become clinically symptomatic very early in the course of the disease. We also found that pilocytic astrocytomas of the brainstem are surprisingly not such a benign disease as supported by many authors [17-19] with rapid progression especially in the first 20 months after diagnosis and stabilization in survival thereafter (see Figure 1a). These data are also supported by Stuer [20] who observed after a median follow-up of 55 months 30% tumor recurrence and 18% deaths. Another surprising result was the lack of pure oligodendroglial brain stem gliomas and the low proportion of oligoastrocytic tumors. Interestingly in the series of Guillamo 25% of biopsied brainstem gliomas were oligodendrocytic or mixed gliomas.

Established prognostic factors in the literature are age, duration of symptoms, KPS, contrast enhancement, MRI “necrosis”, histology and location in the pons and medulla, mainly based on univariate analysis [2,3,21,22]. We confirmed the strong prognostic impact of KPS and age in multivariate analysis and the positive effect of radiation therapy as the cornerstone of therapeutic measures on overall survival (therapy reduces the risk of death by a factor of 0.4). The value of chemotherapy in low and high grade brainstem gliomas is still undefined. Efficacy of different chemotherapeutic agents (temozolomide, nitrosureas or platinum based chemotherapeutic protocols) is currently unproven. At relapse a wide variety of chemotherapeutic agents are used which included BCNU [1,2-bis(2-chloroethyl)-1-nitrosourea], BCNU - procarbazine, CCNU [1-(2-chloroethyl)-3-cyclohexyl-1-nitrosourea] -procarbazine-vincristine (PCV), carboplatin, carboplatin-VP16, carboplatin-VP16-ifosfamid, ifosfamid, procarbazine-VP16, temozolomide, CCNU, vincristine, irinotecan, cisplatin and temozolomide, ACNU and procarbazine. Effectiveness of these protocols were limited: Guillamo [3] reported a radiological response rate of 7% three months after onset of chemotherapy and clinical improvement lasting longer than 6 months in 15% of patients and Samlaggi [4] described a temporary clinical and radiological stabilization in 22% of patients after chemotherapy.

Alternative therapeutic strategies like interstitial radiosurgery with implantation of iodine-125 seeds and application of anti-angiogenic drugs like bevacizumab have to be also considered in the therapeutic concept. Mundinger treated in a series of 89 low grade brainstem gliomas 55 patients with stereotactic brachytherapy. 29 patients received iodine-125 seeds with a 5 year survival rate of 54.8% and 26 patients received iridium 192 seed with a 5 year survival rate of 26.9% in comparison to 5 year survival rate of 14.7% in patients who underwent only biopsy [23]. Ruge recently published a series of 47 patients with inoperable focal brainstem gliomas WHO grades I and II treated by stereotactic implantation of iodine-125 seeds with a 5-year overall survival rate of 97.4 ±2.6% [24].

Survival rates of interstitial radiosurgery are therefore at least comparable to external beam radiotherapy with reported 5-year survival rates between 45%-58% [1,2] and both methods should be evaluation against each other in prospective randomized trials.

Reports about the use of antiangiogenic substances in the literature are rare. Besides two case reports [25,26] a small series of 3 patients [27] showed the effectivness of bevacizumab as a salvage therapy for progressive brainstem gliomas with improvement of clinical condition, reduction of daily dexamethasone dosage and radiological response.

## Conclusion

Adult brain stem gliomas present with a wide variety of neurological symptoms and neuroradiological differential diagnoses. Stereotactic biopsy is the procedure of choice to obtain a histopathological diagnosis. Prognosis of high-grade gliomas resembles its supratentorial counterparts whereas low-grade gliomas of the brainstem have a worse prognosis compared to the actual literature. Cornerstone of therapy remains radiation and alternative strategies like interstitial radiosurgery, chemotherapy or antiangiogenic drugs need to be further explored, ideally in the context of molecular profiling for common alterations such as 1p/19q codeletion, *MGMT* promoter methylation and *IDH* mutation.

## Competing interests

The authors declare that they have no competing interests.

## Authors’ contribution

TR analyzed the data and created the first draft of the article, AK collected and analyzed the data, MT and GN were involved in conception and design of the study, GN critically reviewed the first draft, BH and ML performed statistical analysis and interpretation of data, and all authors approved the final draft.

## Pre-publication history

The pre-publication history for this paper can be accessed here:

http://www.biomedcentral.com/1471-2407/14/115/prepub
